# Supplementary material for: Multi-Omics Integration to Reveal the Mechanism of Sericin Inhibiting LPS-Induced Inflammation
Source: Int J Mol Sci. 2022 Dec 23;24(1):259. doi: 10.3390/ijms24010259 (PMC9820220; doi:10.3390/ijms24010259)
Supplement: Supplementary file 1 [file ijms-24-00259-s001.zip › Supplementary Figure Legends.pdf]

Supplementary Figure legends

Figure S1. (A) The molecular weight of sericin samples. (B-D) Antioxidant effect of sericin samples. The ability to scavenge ABTS (B) and DPPH (C) free radicals and reduce Fe^{3+} (D) of 13.5 mg/mL sericin samples. (E-G) Antioxidant effect of SS2, the sample with the highest purity of sericin. ***$p < 0.001$.

Figure S2. The MA plot and Volcano plot of the differential expressed genes among L/WT (A), L/LS (B), LS/S (C), and S/WT (D). Red points mean differently expressed genes (logFC >=2 or lg(FDR >5)).

Figure S3. (A) Enriched pathways were organized into a network with edges connecting overlapping gene sets. (B) The heatmap of the relationship between genes set and pathways.

Figure S4. (A) Examples of genes whose expression was activated by the addition of LPS but repressed by sericin. (B) Bar graphs represented quantitative analysis of WB bands by Image J. **$p < 0.01$, ***$p < 0.001$, ****$p < 0.0001$.
