# Supplementary figures and images for: Multi-Omics Integration to Reveal the Mechanism of Sericin Inhibiting LPS-Induced Inflammation
Source: Int J Mol Sci. 2022 Dec 23;24(1):259. doi: 10.3390/ijms24010259 (PMC9820220; doi:10.3390/ijms24010259)

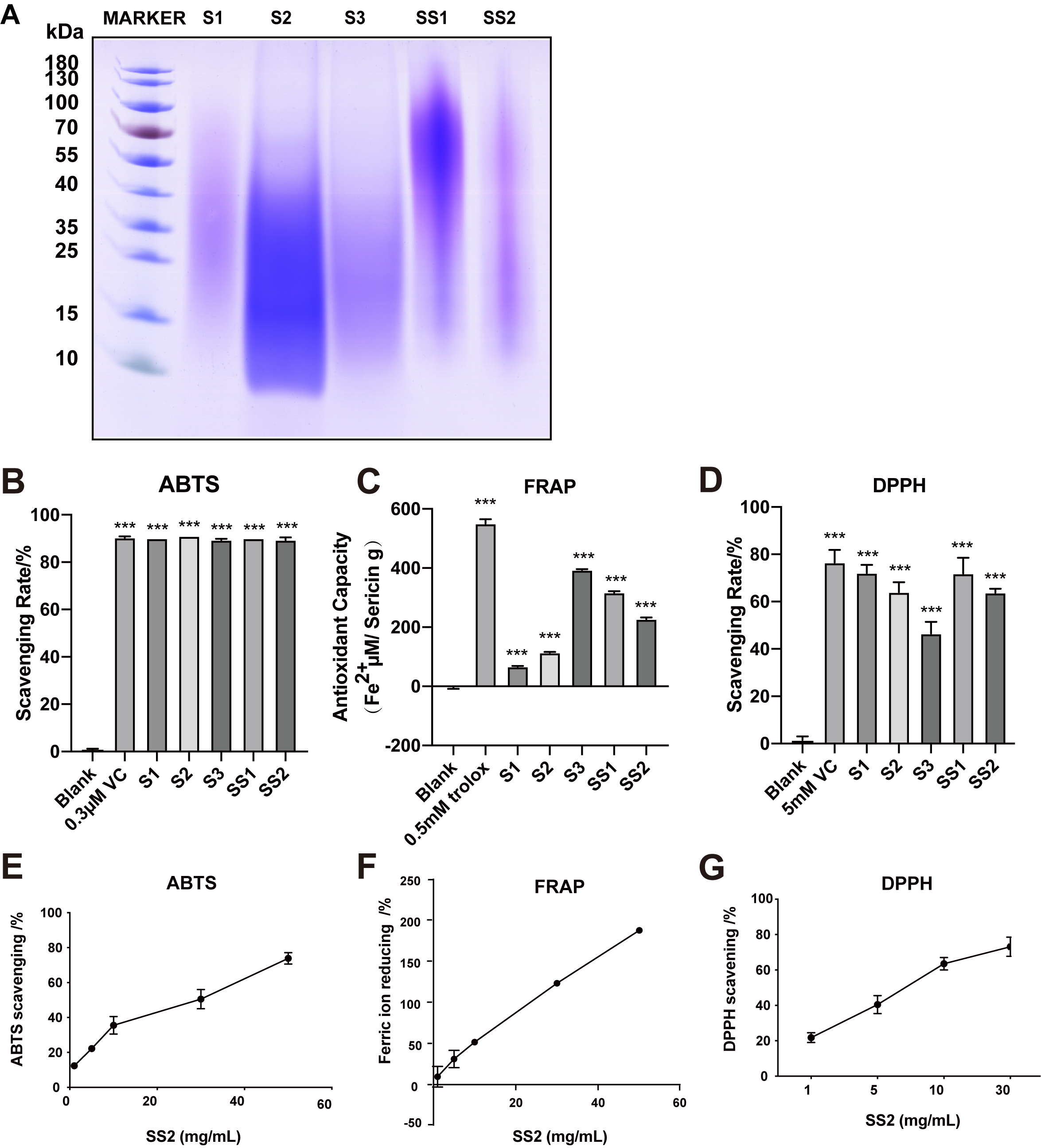

Supplement: Supplementary file 1 [file ijms-24-00259-s001.zip › Figure S1.tif]

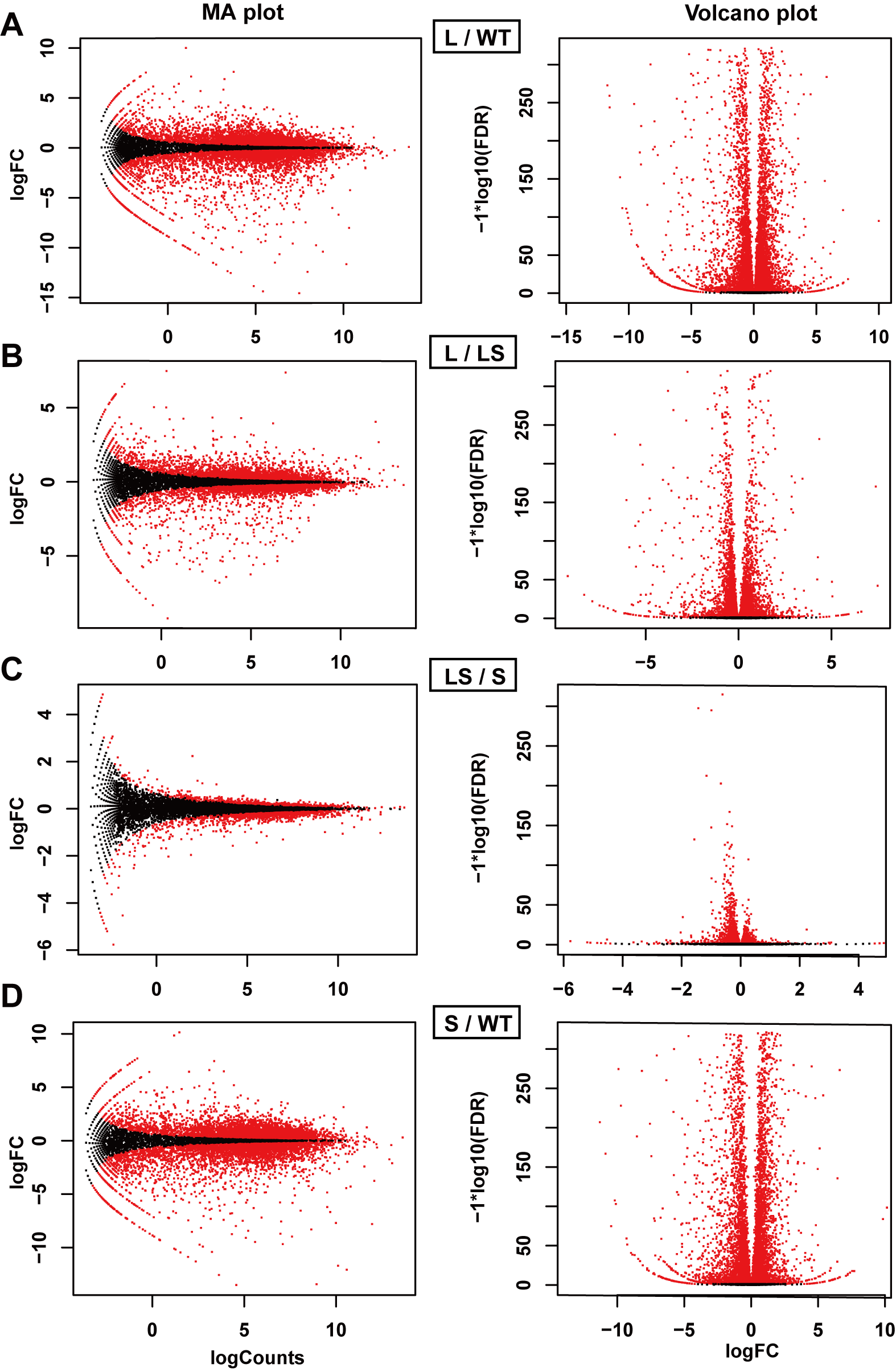

Supplement: Supplementary file 1 [file ijms-24-00259-s001.zip › Figure S2.tif]

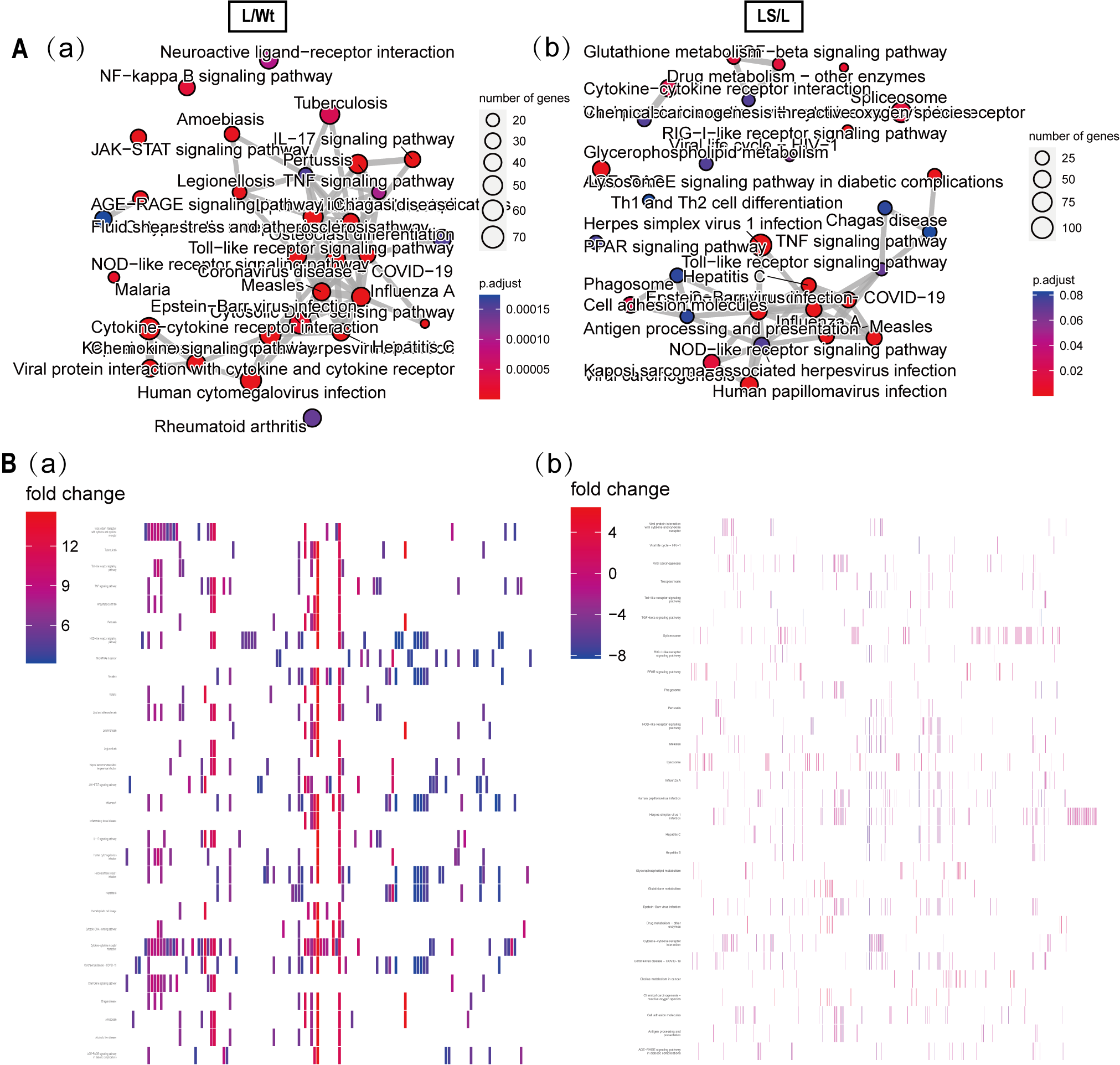

Supplement: Supplementary file 1 [file ijms-24-00259-s001.zip › Figure S3.tif]

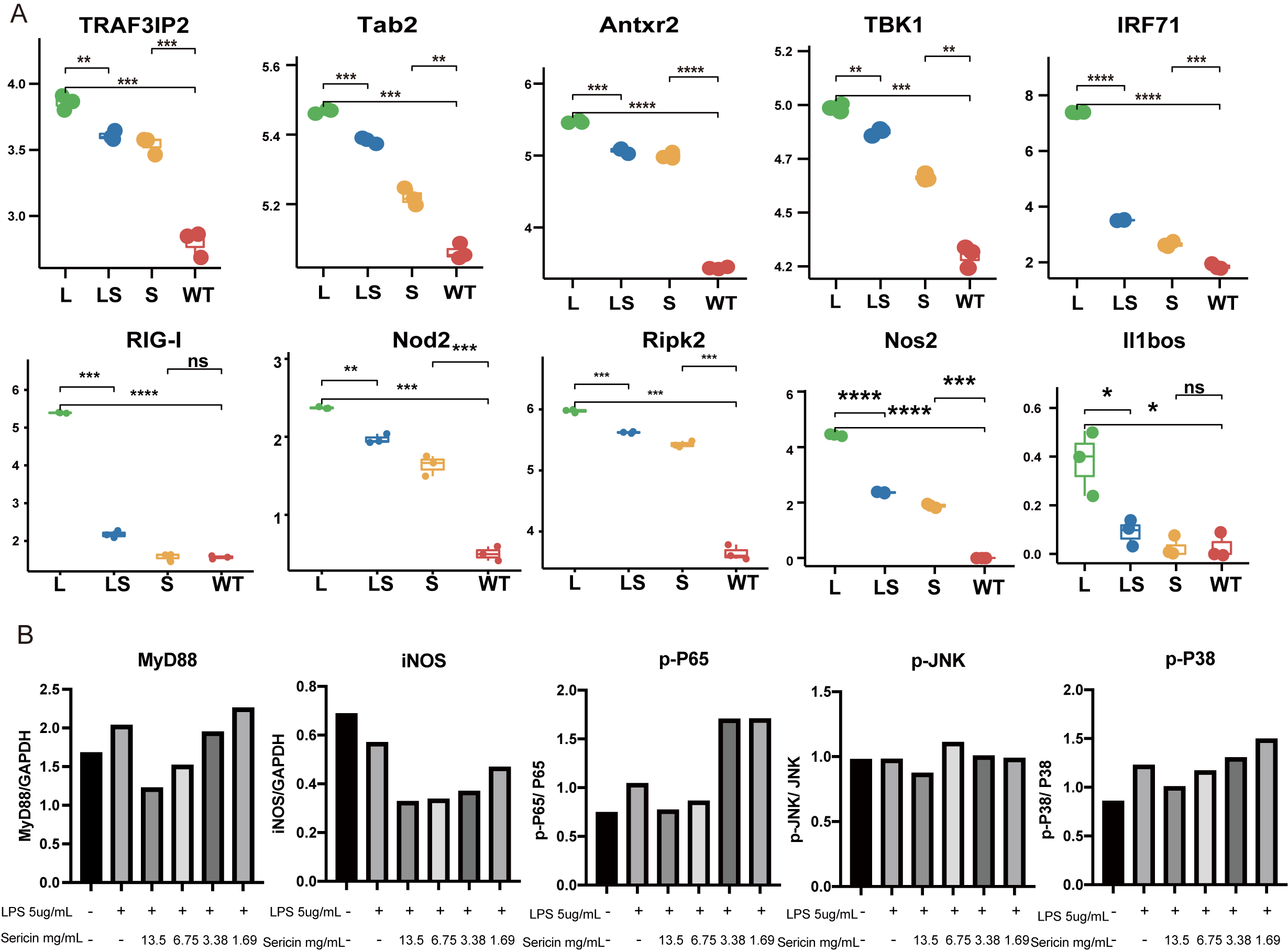

Supplement: Supplementary file 1 [file ijms-24-00259-s001.zip › Figure S4.tif]
